# Preventing and lessening exacerbations of asthma in school age children associated with a new term: pleasant

**DOI:** 10.1186/1745-6215-14-S1-P17

**Published:** 2013-11-29

**Authors:** Steven Julious, Michelle Horspool

**Affiliations:** 1 University of Sheffield, Sheffield, UK

## 

We will present the PLEASANT trial: a relatively novel cluster randomised cohort clinical trial in primary care where GP practices (140) are randomised to a NHS delivered public health intervention or usual care. The outcome data is routine clinical data collected through the Clinical Practice Research Datalink.

The motivation for PLEASANT is a pronounced increase in the number of unscheduled visits to the doctor, by school aged children with asthma around the return back to school in September. This increase is preceded by a drop in the number of prescriptions administered in August. It is possible therefore that children might not be taking their medication as they should just prior to the challenge they get on return back to school from mixing with children when they picking up infections which might affect their asthma. Indeed children with asthma are approximately twice as likely to have unscheduled medical contacts compared to non-asthmatic children in September following return to school.

In the trial school aged children with asthma will be randomised to receive a letter from their GP or usual care. The letter will be sent prior to the start of school holidays reminding of the importance of medication adherence. The aim is to reduce exacerbations of asthma and unscheduled contacts, in school aged children.

Recruitment of sites started in Jan 2013. We will describe the PLEASANT trial and the merits of the design chosen. We will also present results on factors which influence recruitment of centres into a trial.

